# Rebound or Entrainment? The Influence of Alternating Current Stimulation on Individual Alpha

**DOI:** 10.3389/fnhum.2019.00043

**Published:** 2019-02-12

**Authors:** Linus Haberbosch, Sein Schmidt, Andreas Jooss, Arvid Köhn, Leonard Kozarzewski, Maria Rönnefarth, Michael Scholz, Stephan A. Brandt

**Affiliations:** ^1^Department of Neurology, Charité—Universitätsmedizin Berlin, Berlin, Germany; ^2^Neural Information Processing Group, University of Technology Berlin, Berlin, Germany

**Keywords:** alternating current stimulation, neuromodulation, alpha rhythm, rebound, entrainment, phosphenes

## Abstract

Alternating current stimulation (ACS) is an established means to manipulate intrinsic cortical oscillations. While working towards clinical impact, ACS mechanisms of action remain unclear. For ACS’s well-documented influence on occipital alpha, hypotheses include neuronal entrainment as well as rebound phenomena. As a retinal origin is also discussed, we employed a novel form of ACS with the advantage that it specifically targets occipital alpha-oscillations *via* retinofugal pathways retinofugal ACS (rACS). We aimed to confirm alpha-enhancement outlasting the duration of stimulation with 10 Hz rACS. To distinguish entrainment from rebound effects, we investigated the correlation between alpha peak frequency change and alpha-enhancement strength. We quantified the alpha band power before and after 10 Hz rACS in 15 healthy subjects. Alpha power enhancement and alpha peak frequency change were assessed over the occipital electrodes and compared to sham stimulation. RACS significantly enhanced occipital alpha power in comparison to sham stimulation (*p* < 0.05). Alpha peak frequency changed by a mean 0.02 Hz (± 0.04). A greater change in alpha peak frequency did not correlate with greater effects on alpha power. Our findings show an alpha-enhancement consistent with studies conducted for transcranial ACS (tACS) and contribute evidence for a retinal involvement in tACS effects on occipital alpha. Furthermore, the lack of correlation between alpha peak frequency change and alpha-enhancement strength provides an argument against entrainment effects and in favor of a rebound phenomenon.

## Introduction

Non-invasive brain stimulation (NIBS), including transcranial electric stimulation (tES; Paulus, 2011), has shown impressive effects ranging from short changes in neural activity to long lasting recovery maximization following neural injury (Hallett, 2005; Talelli and Rothwell, 2006; Hummel et al., 2008; Sandrini and Cohen, 2013). A novel form of NIBS is transcranial alternating current stimulation (tACS), an oscillatory stimulation technique associated with psychophysical changes (Antal et al., 2008; Kanai et al., 2010; Wach et al., 2013), enhancement of working memory, learning, and long-term memory formation (Marshall et al., 2006; Kuo and Nitsche, 2012; Santarnecchi et al., 2013), as well as clinical improvements including tumor growth slowing (Kirson et al., 2007) and tremor suppression in patients with Parkinson’s disease (Brittain et al., 2013). Despite these encouraging results, little is known about the mechanism of action (Zaghi et al., 2010). Due to this lack of knowledge tACS may consequently be in danger of facing, similar to transcranial direct current stimulation (tDCS; Horvath et al., 2015), the challenges of large effect variability and poor result reproducibility (Zaghi et al., 2010; Feurra et al., 2013; Wach et al., 2013). There is also evidence that the effects of tACS are at least in part due to retinal stimulation (Schutter, 2016). To efficiently and efficaciously apply tACS, especially in the visual system, it critically important for its users to further investigate the retinal as well as cortical mechanisms of action behind the observed effects.

In general, the effects of AC stimulation have been mainly attributed to synchronization of neural oscillations (Antal et al., 2008; Paulus, 2011; Herrmann et al., 2013), with most effects reported on alpha (α) oscillations (8–12 Hz; Antal et al., 2008; Helfrich et al., 2014; Vossen et al., 2015), the dominant frequency of the visual cortex (Klimesch, 1999). As the mechanism of action is unknown and effects of photic stimulation (Photic Driving; Walker et al., 1944) as well as AC stimulation on α oscillations *via* the retinofugal (visual) pathway are well-known (Brindley, 1955; Grützner et al., 1958; Sakamoto et al., 1993; Rager and Singer, 1998; Schmidt et al., 2013a), a better understanding of the underlying neurophysiology of alternating current stimulation (ACS) effects on oscillations should help address these issues.

Hypotheses concerning the mechanism of action for ACS synchronization effects include *entrainment* of neural oscillations as well as *rebound* effects. While entrainment describes the synchronization of one oscillator to another (Ermentrout and Rinzel, 1984), rebound is defined as an increase in excitability typically following inhibition (Perkel and Mulloney, 1974).

Several studies have shown frequency-specific effects on perceptual or cognitive task performance, ascribed to a potential frequency entrainment during or shortly after a single train of rhythmic stimulation (Klimesch et al., 2003; Marshall et al., 2006; Kanai et al., 2008; Zaehle et al., 2010). Entrainment requires an increase of spectral power as well as phase- and frequency-lock of a neural oscillation to an external stimulus (Vossen et al., 2015).

Phase-lock has been reported during stimulation (Helfrich et al., 2014; Ruhnau et al., 2016), although electrical stimulation artifacts in EEG and MEG aggravate the acquisition of reliable neurophysiological data (Soekadar et al., 2013; Helfrich et al., 2014). Newly presented data by Noury et al. suggests that these artifacts may be mistaken for entrainment effects (Noury et al., 2016).

Consequently, most reports present spectral power enhancement only after the cessation of ACS (Zaehle et al., 2010; Schmidt et al., 2013a; Helfrich et al., 2014; Vossen et al., 2015). This offers the hypothesis of a rebound effect as an alternative explanation. Classical post-inhibitory rebound is an increase in excitability following inhibition, generating responses ranging from threshold lowering up to a train of impulses (Perkel and Mulloney, 1974). This, considered for α-pacemaker neurons, would also result in an increase of spectral power after stimulation. Such an increase would occur at an intrinsic frequency and without a phase-lock (Perkel and Mulloney, 1974). Neural rebound effects after stimulation have been found in animal models (Pape and McCormick, 1995; Tong et al., 1996; Huang et al., 2008) as well as in the human brain (Fuggetta et al., 2005; Brignani et al., 2008; Manganotti et al., 2012).

The primary goal of this study was to examine the mechanisms of action behind ACS effects on occipital α oscillations. We therefore investigated a possible contribution of retinal stimulation to the observed α enhancement by employing a periorbital montage. Furthermore, to address the differentiation between neural entrainment and rebound effects after AC stimulation, any frequency peak shift in endogenous α rhythms towards the stimulation frequency was investigated. The presence of such a frequency peak shift would indicate an entrainment, while its absence would provide evidence in favor of a rebound phenomenon.

We decided on the α frequency band as a target, as ACS has shown robust effects in this frequency range (Kanai et al., 2010; Helfrich et al., 2014; Vossen et al., 2015). Cortical α is associated with numerous perceptional processes (Surwillo, 1961; VanRullen and Koch, 2003; Mathewson et al., 2009; Ai and Ro, 2014; Lange et al., 2014; Cecere et al., 2015; Samaha and Postle, 2015) as well as cognitive performance (Klimesch, 1999; Klimesch et al., 2003; Hanslmayr et al., 2005; Zoefel et al., 2011) and characterized by a peak in spectral analysis, the individual α frequency (IAF; Klimesch et al., 2007), which presents an optimal opportunity to investigate synchronization to external stimulation. The Berger effect, describing the repression of the highest physiological occipital α in a subject *via* opening of the eyes (Berger, 1929), presents further opportunities. Firstly, we can employ our stimulation in an eyes open condition and expect little interference from intrinsic α. Furthermore, the measurement of high occipital α during an eyes closed baseline condition can serve as a reference for effect size, since it allows us to compare exogenous stimulation effects to the highest intrinsically generated α power.

Retinal contribution to tACS effects is part of an ongoing discussion (Schwiedrzik, 2009; Paulus, 2010; Schutter and Hortensius, 2010). A recent review by Schutter (2016) suggested that phosphenes might be involved in tACS α-enhancement in the visual system.

To further investigate this and effectively target α oscillations in the visual cortex, we employed a periorbital application type of ACS termed retinofugal ACS (rACS). This technique has been applied aiming for vision restoration in a therapeutic regimen called repetitive transorbital ACS (rtACS; Gall et al., 2010). It utilizes signal transmission along well-defined retinofugal tracts terminating predominantly in cortical visual areas (Gray and Singer, 1989) and offers a comparatively focal (Peterchev et al., 2012) method to investigate ACS effects on intrinsic frequencies in the well-circumscribed visual system (Gall et al., 2011; Schmidt et al., 2013a).

## Materials and Methods

### Participants

We stimulated 15 healthy volunteers in the rACS group (eight female, seven male, mean age 23.9 ± 2.5) as well as in the sham group (four female, 11 male, mean age 25.8 ± 5.3). The subjects were interviewed prior to experimentation regarding their state of health and gave written informed consent. We applied established exclusion criteria for NIBS (Brunoni et al., 2012), and added evidence for photophobia and photosensitive epilepsy. All protocols conformed to the Declaration of Helsinki, and were approved by the Ethics Committee of the Charité—Universitätsmedizin Berlin (“Ethikkommission der Charité—Universitätsmedizin Berlin”). This study adheres to the principles of good scientific practice of the Charité—Universitätsmedizin Berlin (“Grundsätze der Charité zur Sicherung guter wissenschaftlicher Praxis”).

### Stimulation

RACS was applied by a multi-channel low-voltage stimulation device certified for clinical use, which delivered voltage-controlled weak periorbital sinusoidal current over four individual periorbital electrodes respectively (NextWave, Eyetronic, Germany). The four superficial active stimulating electrodes (sintered Ag/AgCl ring electrode, Easycap, Germany) were contained in foam-padded stimulation goggles and bilaterally made skin contact *via* small felt buffers (0.35 cm^2^) superior and inferior to the eye (“periorbital”). The passive electrode (rectangular electrode, 30 × 30 mm polished stainless steel) was fastened on the back of the neck at the midline relative to the occipital poles.

The electrical impedance of the four stimulating electrodes was measured for four different frequencies (5 Hz, 10 Hz, 15 Hz and 20 Hz) and held below 15 kΩ. This measurement at different frequencies was done as part of the innate safety procedure of the CE-certified stimulation device. Phosphene thresholds, defined as the current intensity when participants first subjectively perceived phosphenes, were determined employing an ascending method of limits (Herrick, 1967) provided by the NextWave software.

In the rACS group, stimulation was applied with bandwidth restricted to 10 Hz at an amplitude of 120% phosphene threshold (resulting in a mean 354.15 μA ± 50.6 peak-to-peak amplitude) and delivered in six 30 s blocks followed by 30 s pauses each. Solely 10 Hz as stimulation frequency was chosen to stay close to the critical α-eigenfrequencies of our subjects.

In the sham group, six blocks of a 5 s ramp-up/ramp-down DC stimulus followed by 25 s signal silence was applied at 120% phosphene threshold (resulting in a mean 292.10 μA ± 68.9 peak-to-peak amplitude), again followed by 30 s pauses each. During the signal silence, the stimulator remained switched on while no current was delivered. The sham signal was designed to match initial skin sensations known from other forms of NIBS (Siebner et al., 2004) as well as rACS phosphene perception.

### EEG

EEG measurements were performed during the sessions using a 32-electrode EEG cap (Waveguard EEG caps, ANT BV, Enschede, Netherlands), according to the 10–20-system, with impedances kept below 10 kΩ. Data acquisition was carried out in a shielded room without natural light in the electrophysiological unit of the neurological department.

Two minutes of baseline EEG with four conditions were recorded prior to stimulation: 30 s eyes open, 30 s eyes closed and 30 s blinking followed by 30 s of muscle artifact production. To avoid artifacts, the subjects were told to focus a fixed point on a white surface in 1 m distance for the duration of the experiment. Moreover, the experimenter controlled the eyes open/closed conditions and reminded the subjects to follow the instructions and keep alert if necessary.

The EEG data was imported into MATLAB R2014a (The MathWorks Inc, Natick, MA, USA) and analyzed using the FieldTrip toolbox (Oostenveld et al., 2011). We segmented the EEG signals in 25 s-intervals for the eyes-open (EO) baseline, the eyes-closed (EC) baseline and the EO post-stimulation (Post-Stim) condition, including data from 2.5 s after stimulation cessation to 2.5 s before the beginning of a new stimulation block. Remaining artifacts, especially blinking and muscle twitches, were excluded *via* visual artifact rejection for each trial and channel. The blinking and muscle artifact conditions run prior to baseline EEG served as a decision support in borderline cases. EEG signals were referenced against the common average and filtered (Band-Pass Filter from 2 to 70 Hz with filter-slope 24 dB/oct). Following resampling of the data from originally 2,000–500 Hz to streamline and accelerate data analysis, a fast fourier transformation (FFT) with a discrete prolate spheroidal sequences (DPSS) multitaper determined the bandwidth-specific power (mV^2^). The sliding time window was set to 0.5 s, while the frequency analysis window was set to 0.04 Hz. The frequency with the highest resulting α power was defined as the IAF/α peak. For illustration, we calculated the EEG-power spectra grand average during EC and post-stimulation and baseline-corrected them by dividing it by the EO baseline grand average. Topographical plots of these values were then generated with FieldTrip and smoothed using a moving average filter with a span of two.

After this, the mean α power over the spectrum 8–12 Hz for each electrode and stimulation block was calculated. We then confirmed that there was no significant correlation between stimulation block number and α power increase in either the sham (*r* = 0.02, *p* = 0.83) or the rACS condition (*r* = −0.08, *p* = 0.45) *via* Spearman’s rho to ensure the absence of an alpha power ramp-up effect over consecutive trains of stimulation. Consecutively, we calculated the mean α power and peak frequency over all six stimulation blocks for each electrode, preparing the data for statistical analysis.

### Statistics

To statistically investigate the effect of rACS on occipital alpha, we compared the mean spectral α (8–12 Hz) α power for rACS and Sham. Due to the lack of normal distribution determined by the Shapiro-Wilk test, we ln+1-transformed the data. Normal distribution and homoscedasticity of the data were then confirmed *via* the Shapiro-Wilk and Levene’s test respectively. We then calculated a repeated measure analyses of variance (ANOVA) with the dependent variable “α power”, consisting of the levels (EO Baseline, EC Baseline and EO Post-Stim) as well as the fixed factor “group.”

For our analysis of α power change, we baseline-corrected the ln-transformed EO Post-Stim α power data by dividing it by the EO Baseline and subtracting 1. After confirming normal distribution and homoscedasticity, we performed an ANOVA with “group” as fixed factor and baseline-corrected ln-transformed “alpha power” as dependent variable.

To gain insight on a possible frequency shift after rACS, the effect of rACS of the distance of α peak frequency to stimulation frequency was analyzed with an univariate ANOVA after confirming normal distribution of the data *via* Shapiro-Wilk test. A possible correlation between distance of individual α to stimulation frequency and α-power change was also assessed, again using the Spearman correlation coefficient.

*P*-values of ≤ 0.05 were considered significant. All analyses were performed using IBM SPSS Statistics, Version 19.0.0.1 (IBM, Armonk, NY, USA). Spectral power bar plots as well as spectral α peak box- and scatter plots were created using GraphPad Prism version 7.02 for Windows (GraphPad Software, La Jolla, CA, USA^1^).

## Results

### Stimulation Parameters

An average phosphene threshold at 290.24 μA (± 44.16) for the rACS and 243.33 μA (± 57.07) for the sham group, impedances at 12.57 kΩ (± 1.8) and 11.73 kΩ (± 2.7), as well as an average peak-to-peak amplitude of 354.15 μA (± 50.6) for rACS and 292.00 (± 68.5) μA for sham were noted. We additionally calculated the effective (root mean square) amplitude, resulting in a mean 250.41 μA (± 47.7) for rACS and 168.58 μA (± 39.5) for sham. The current density amounted to a mean 0.71 mA/cm^2^ (± 0.13) for rACS and a mean 0.48 mA/cm^2^ (± 0.11) for sham.

### Alpha Power Enhancement After rACS

We found a spectral α power enhancement over the occipital scalp area after rACS, with subjects showing significantly greater α power increase after rACS as compared to sham. The enhancement was comparable in size to the one found in the EC condition.

**Figure 1:**
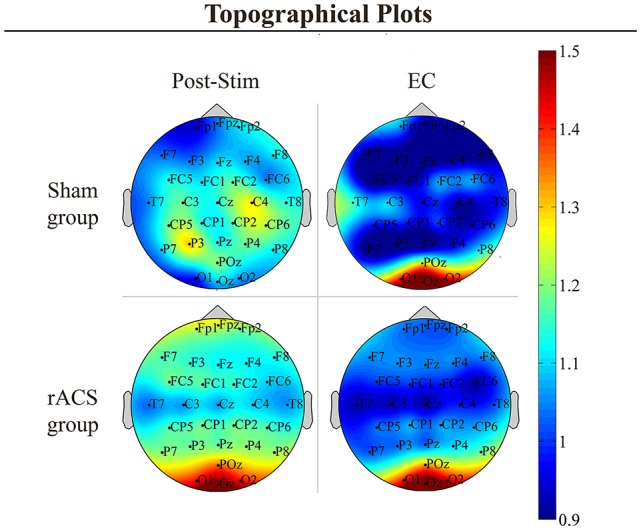
Topographical plots. Topographical plots of the α power change [divided by eyes open (EO) Baseline] in the conditions post-stimulation (Post-Stim) and eyes closed (EC) Baseline as well as the retinofugal alternating current stimulation (rACS)- and Sham group. Color spectrum ranges from 0.9 to 1.5. In the EC and rACS conditions, α increase is most prominent across the occipital electrodes O1, Oz and O2.

Topographical plots of the frequency grand average (Figure 1) showed a strong focus of the overall α-power enhancement after rACS and during EC around the occipital scalp area. Additionally, a diffuse increase in 8–12 Hz spectral power can be observed after sham and rACS, with foci in the frontal and centroparietal scalp area after rACS and in the central scalp area after sham.

**Figure 2:**
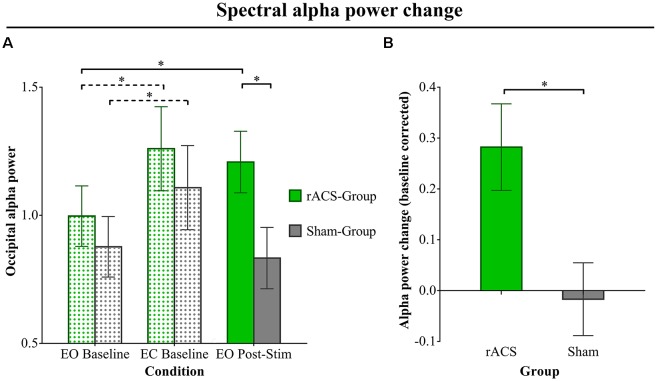
Spectral alpha power change. **(A)** Ln+1-transformed spectral α power over the occipital electrodes O1, Oz and O2 for the rACS group (green) and the Sham group (gray) in three conditions: eyes open (EO) Baseline, eyes closed (EC) Baseline and EO Post-Stim. Baseline condition bars are dot patterned, while the Post-Stim bars are plain. Error bars depict standard error of the mean. The EC Baseline shows a significant increase in α power compared to EO Baseline in both groups, replicating the Berger effect (indicated by dashed lines). The EO Post-Stim condition α in the rACS group is significantly higher than the Sham EO Post-Stim α, as well as significantly higher than its (rACS Group) EO Baseline (indicated by solid lines), showing a clear stimulation effect of rACS on occipital α. **(B)** Mean baseline-corrected α power change (EO Post-Stim/EO Baseline −1) over the occipital electrodes O1, Oz and O2 for the sham and rACS groups. Post-stimulation rACS is depicted as green, post-stimulation sham as gray. A value of 0 represents no change from baseline α power. Error bars depict standard error of the mean. The rACS group shows a significantly stronger increase in α power compared to the sham group. Significant differences (*p* < 0.05) are marked with an asterisk *.

Mean ln+1-transformed EO Baseline α power (Figure 2A) amounted to 0.99 (± 0.13) for the rACS group and 0.88 (± 0.10) for the Sham group. In the EC Baseline condition, we found a mean α power of 1.26 (± 0.17) in the rACS and 1.11 (± 0.16) in the Sham group. Alpha power in the EO Post-Stim condition amounted to a mean 1.21 (± 0.14) in the rACS and a mean 0.83 (± 0.09) in the Sham group. The repeated measure ANOVA resulted in a significant main effect for mean α power (*F*_(2,27)_ = 8.99, *p* = 0.001) as well as interaction between the stimulation type and mean alpha power (*F*_(2,27)_ = 4.02, *p* = 0.03). Bonferroni-corrected pairwise comparisons showed a significantly higher alpha power in the rACS Post-Stim condition compared to Sham (*p* = 0.036), but no significant differences in the baseline (*p* = 0.48) and EC (*p* = 0.52) conditions. The rACS group also showed significantly higher EO Post-Stim α power compared to EO Baseline (*p* = 0.01), whereas the Sham group showed no significant difference in this regard (*p* = 0.99). Furthermore, EC Baseline α power was significantly higher than the EO Baseline in both groups (rACS: 0.02, Sham: 0.05), replicating the Berger-effect. There was no significant difference between rACS group EO Post-Stim α and EC Baseline (*p* = 1.00), indicating a physiologically plausible α increase.

Baseline-corrected α power (change; Figure 2B) amounted to a mean −0.02 (± 0.07) for EO Post-Stim Sham and +0.28 (± 0.09) for EO Post-Stim rACS. The ANOVA showed a significantly higher alpha power change after rACS compared to Sham (*F*_(1,28)_ = 7.139, *p* = 0.01).

### Individual α Frequency Shift

**Figure 3:**
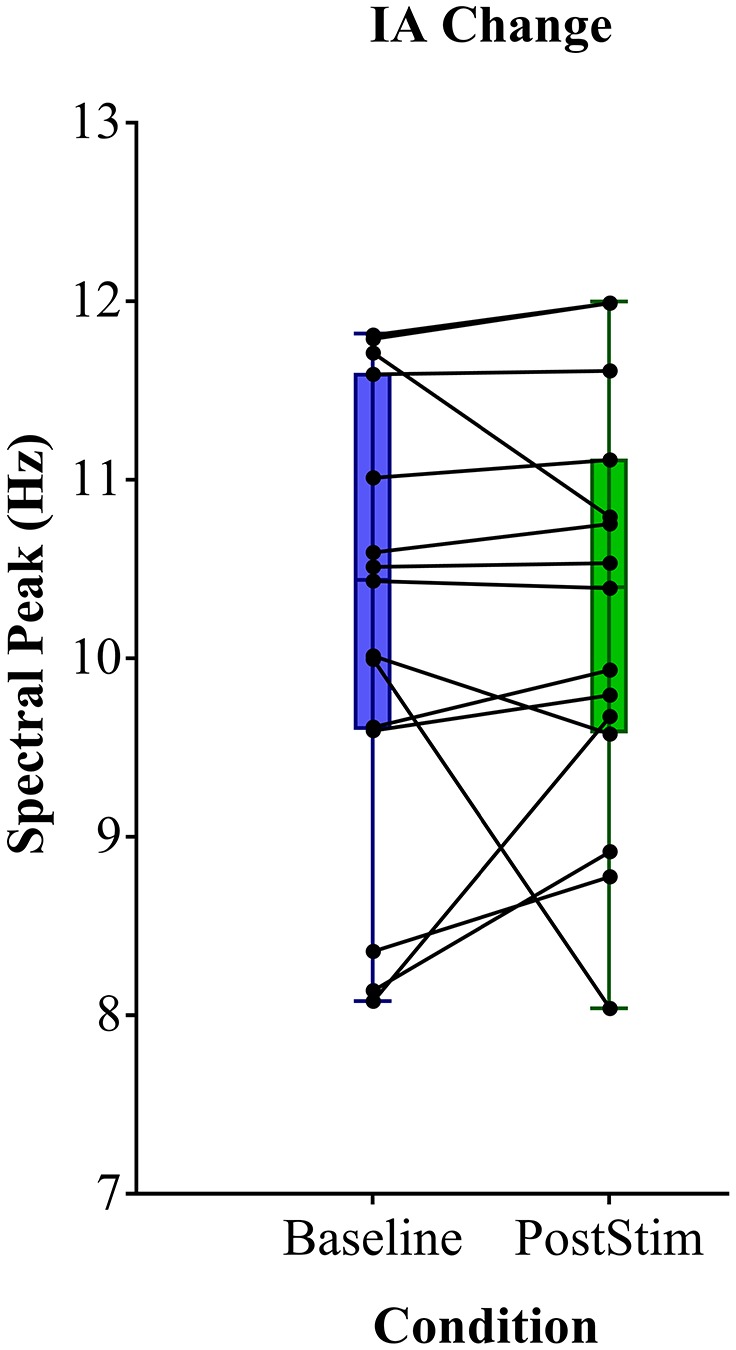
Mean spectral peak analysis. Individual α peak frequency before (blue) and after (green) rACS. Black points and lines depict individual subjects. No consistent shift of the individual α peak to stimulation frequency can be observed.

We found no significant shift of the individual α peak frequency after rACS compared to baseline. The spectral peak of the α-band amounted to a mean 10.22 Hz (± 1.29) prior to rACS and a mean 10.26 Hz (± 1.17) after rACS (Figure 3). The mean absolute distance to stimulation frequency (10 Hz) amounted to 1.05 Hz (± 0.73) before and 0.97 Hz (± 0.67) after application of rACS. There was no significant effect of rACS on the distance of α peak frequency to stimulation frequency (*F*_(1,28)_ = 0.009, *p* = 0.93) as assessed by an univariate ANOVA. This indicates the lack of an overall frequency lock of intrinsic α to stimulation frequency.

Moreover, the proximity of IAF to the stimulation frequency of 10 Hz did not correlate to a greater increase in α-power (*r* = 0.04, *p* = 0.88) employing Spearman’s rho. This provides evidence against accidental frequency-locked stimulation leading to enhanced occipital α.

## Discussion

The effects of 10 Hz rACS on neural oscillations showed the following characteristics: (1) 10 Hz stimulation resulted in an enhancement of α-power; (2) the post-stimulation α-peak did not significantly differ from baseline IAF; and (3) there was no significant correlation between α-power and proximity to stimulation frequency. These findings are not consistent with the hypothesized entrainment effects. Since entrainment of neural oscillations is the most proposed mechanism of action for tACS effects (Helfrich et al., 2014), an in-depth discussion of these conflicting findings is required.

### Enhancement

The specific enhancement of α-power over the occipital electrodes after 10 Hz rACS is consistent with studies conducted for tACS (Antal et al., 2008; Zaehle et al., 2010; Helfrich et al., 2014; Vossen et al., 2015). While a diffuse increase in 8–12 Hz spectral power in the frontal and centroparietal scalp can be observed after rACS, the distribution of the enhancement was comparable to that of the highest physiological α in the EC condition. Additionally, it did not significantly differ from EC regarding effect size, showing a physiologically plausible increase in synchrony. A similar α-enhancement outlasting stimulation duration has already been reported after 10 Hz audiovisual (Rosenfeld et al., 1997) and photic stimulation (Sakamoto et al., 1993; Spaak et al., 2014). This supports the hypothesis of Schutter (2016) that retinal stimulation may induce neural effects similar to tACS. Retinal phosphenes induced by tACS and their possible role in ACS effects of neural oscillations are subject of a long ongoing discussion.

Early work by Kanai et al. (2008) showed phosphene elicitation during occipital tACS in a frequency- and illumination-dependent manner. While this was interpreted as cortical stimulation, others proposed a retinal origin. The higher sensitivity of the retina and an experimentally confirmed occipital-to-frontal threshold decrease supported this hypothesis (Schwiedrzik, 2009; Schutter and Hortensius, 2010). Interestingly, moving the electrode from the occipital area towards the retina did not change the latency of phosphene perception (Kar and Krekelberg, 2012). The effects of tACS over the visual cortex may therefore be a result of stimulation along the retinofugal pathway similar to rACS.

Yet, due to the respective montages there should be a magnitude of difference between methods (Peterchev et al., 2012) with rACS inducing the most, traditional occiput-vertex montages intermediate and bilateral or 4 × 1 ring electrode montages inducing the least retinal activation (Paulus, 2010; Neuling et al., 2012; Datta et al., 2013; Laakso and Hirata, 2013).

In sum, we provide further evidence for α-power-enhancement after ACS being induced *via* retinofugal pathway activation.

### Entrainment

Neural oscillators, as the target of tACS, share features of relaxation oscillators and harmonic oscillators (Glass, 2001; Winfree, 2001) and therefore present a stable eigenfrequency as well as synchronization capabilities (Somers and Kopell, 1993; Buzsáki and Draguhn, 2004).

This enables entrainment to an external force resulting in effects that could outlast stimulation duration. Such entrainment effects would present themselves as phase- and frequency-locked to stimulation and have been shown *in vitro* (Fröhlich and McCormick, 2010) and in the animal model (Ali et al., 2013).

We observed no such frequency-lock after rACS.

This lack of a shift towards stimulation frequency is also consistent with several reports of tACS α-enhancement post-stimulation (Zaehle et al., 2010; Helfrich et al., 2014; Vossen et al., 2015). Vossen et al. (2015) have also presented this as strong evidence against entrainment, whereas Helfrich et al. (2014) and Zaehle et al., 2010 focused on ACS effects during stimulation *via* artifact rejection. The lack of a correlation between proximity of IAF to stimulation frequency has also been reported for tACS (Helfrich et al., 2014), even going as far as a negative correlation (Vossen et al., 2015). Both groups have reported these findings as counterintuitive. While this presents an argument against entrainment, one would need reliable recordings during stimulation to falsify this hypothesis.

Still, entrainment effects could produce the results at hand in the following ways.

#### Entrainment During Stimulation

The effects of stimuli on neuronal oscillators are mainly dependent on phase and stimulus amplitude (Glass, 2001). RACS was not applied at IAF and consequently not applied phase-locked as well. Therefore, the stimulus was unlikely to arrive at the opening phase of the target oscillator. This could have been compensated through a higher stimulus amplitude (Ai and Ro, 2014). Although possible for TMS (Thut and Miniussi, 2009), this is unlikely for most types of tES (including rACS), which reach comparatively low current densities at the target site (Poreisz et al., 2007). As we encountered stimulation artifacts as well as a residual stimulation cessation artifact covering the first 100–300 ms after stimulation, an entrainment effect in this time period cannot be reliably investigated and therefore remains a possibility. There are reports presenting such entrainment during stimulation utilizing artifact rejection techniques (Soekadar et al., 2013; Helfrich et al., 2014; Neuling et al., 2017), which are still controversially discussed (Noury et al., 2016).

#### Frequency Pulling

A stable entrainment during stimulation with an external oscillator would also not explain the immediate loss of synchronization to the external oscillator following stimulus cessation found in this study. A possible explanation is a weak coupling during stimulation, resulting in not a frequency lock, but rather a frequency pulling (Cross et al., 2004) with frequent phase walk-throughs (Ermentrout and Rinzel, 1984). Hereby, an oscillator, instead of fully synchronizing, only appeases the frequency of an external stimulus and desynchronizes quickly (Hoppensteadt and Keener, 1982). This can be the result of the external stimulus being too weak or too far removed from the oscillators eigenfrequency (Cross et al., 2004). A short period of synchronization followed by desynchronization would be the result, which has been reported for the neurophysiologically similar photic stimulation (Jin et al., 2000). A polysynaptic recruitment of further neurons after the initial driving could then explain the observed enhancement (Ozen et al., 2010).

#### Subthreshold Modulation

The lack of a frequency appeasement may also be due to the applied stimulation inducing only subthreshold modulation as shown in the rat model (Ozen et al., 2010). This would result in a lower threshold for network-induced membrane voltage fluctuations to generate spikes in a fraction of the neuronal population. The effects of transcranial stimulation, specifically tES, are mostly attributed to such subthreshold modulation (Zaghi et al., 2010). In contrast to the cortical stimulation of tES, rACS stimulated the more excitable retina (Lindenblatt and Silny, 2002) with amplitudes above phosphene threshold. We can therefore assume the generation of action potentials at least in the visual cell (Grützner et al., 1958; Schutter and Hortensius, 2010). Nevertheless, a threshold-lowering effect of rACS would provide an explanation for similar effects on oscillations found after retinal random noise stimulation (RNS; Jooss et al., 2015) through the mechanism of stochastic resonance (Wiesenfeld and Moss, 1995; Gluckman et al., 1996).

### Rebound

As the entrainment-hypothesis delivers no conclusive explanation for the alpha enhancement following ACS, our findings can be interpreted as evidence for a rebound effect.

Rebound firing is a long known mechanism of action for signal transmission in neurons (Creutzfeldt et al., 1962; Grenier et al., 1998). Diffuse electric fields have been shown to promote a rebound-like burst firing compared to direct somatic current injection (Radman et al., 2009), especially after cessation of stimulation (Reato et al., 2010). Several studies presented rebound firing in thalamocortical networks and neurons in response to oscillatory electric stimuli (Destexhe et al., 1996; Birdno et al., 2014; Sakata, 2016). Thalamocortical networks are well-known to be vitally involved in the generation of the α rhythm (Hughes and Crunelli, 2005).

TMS effects on neural oscillations have also been attributed to rebound firing, with studies finding an α- and/or β-enhancement following single TMS-Pulses (Paus et al., 2001; Fuggetta et al., 2005; Brignani et al., 2008). Paus et al. (2001) explained this as a combination of rebound-like firing and recruitment of “idle” neurons. Fuggetta et al. (2005) as well as Brignani et al., 2008 discuss α-rebound as a possible effect of corticothalamic feedback mechanisms. Following thalamic input, primary visual cortex layer 6 neurons are able to deliver antiphasic feedback to the lateral geniculate nucleus (Yousif and Denham, 2007), which in turn may result in burst firing inducing α- or θ-rhythms (Hughes and Crunelli, 2005). This is particularly interesting, as cortical tACS should activate layer 6 neurons as well and might trigger similar rebound mechanisms.

Applying these considerations, the findings reported in this article can be interpreted as follows: a possible initial entrainment of layer 4 and 6 neurons could result in a corticothalamic feedback triggering thalamic rebound firing, which would drive the cortically observed α-rhythm and lead to further polysynaptic recruitment of cortical neurons. The rebound effect could explain an enhancement and stabilization at an intrinsic frequency after neither phase- nor frequency-locked stimulation (Perkel and Mulloney, 1974). This would also sufficiently explain the lack of correlation between proximity of stimulation frequency to IAF and α-power increase also reported by Helfrich et al. (2014). However, due to the lack of recordings during stimulation, we cannot report a period of inhibition preceding a rebound (Perkel and Mulloney, 1974).

Generally, the challenges of the rebound hypothesis lie in frequency-specific psychophysical effects of ACS reported during stimulation (Pogosyan et al., 2009; Joundi et al., 2012) and the findings of photic driving (Walker et al., 1944), especially as we stimulate the retina and the optic nerve.

### Outlook

A combination of entrainment and rebound hypotheses as presented above may prospectively provide an integrative model of ACS effects on the α-rhythm. Investigation of closed-loop phase-locked stimulation (Brittain et al., 2013) as well as better knowledge of psychophysical changes during stimulation could contribute to a deeper understanding of such mechanisms of action.

Users of tACS in the visual system should take note of the accumulating evidence of a retinal contribution, at least to the effects of cortical α power. Furthermore, cortical tACS should activate layer 6 neurons similar to rACS and might therefore trigger the same rebound mechanisms described in this article instead of achieving entrainment effects.

Finally, a thorough study of the effects achieved by frequency-unspecific RNS, especially in combination with tACS, could provide further insight into subthreshold modulation induced by tES. This would be an intriguing step towards the understanding of a common framework utilizing both noise and oscillation in the human brain (Schmidt et al., 2013b).

## Author Contributions

LH, SS, MS and SB conceived and designed the study. LH, SS, LK and AJ carried out data acquisition and analysis. All authors participated in the interpretation of the data. The manuscript was drafted by LH and SS and critically revised by AJ, AK, MR, MS, LK and SB.

## Conflict of Interest Statement

The authors declare that the research was conducted in the absence of any commercial or financial relationships that could be construed as a potential conflict of interest.

## Footnotes

^1^http://www.graphpad.com
